# Sociodemographic inequities in dental care utilisation among governmental welfare recipients in Japan: a retrospective cohort study

**DOI:** 10.1186/s12939-021-01473-8

**Published:** 2021-06-16

**Authors:** Daisuke Nishioka, Keiko Ueno, Shiho Kino, Jun Aida, Naoki Kondo

**Affiliations:** 1 Department of Medical Statistics, Research & Development Center, Osaka Medical and Pharmaceutical University, Osaka, Japan; 2 Department of Health and Social Behavior, Graduate School of Medicine, The University of Tokyo, Kyoto, Japan; 3 Department of Social Epidemiology, Graduate School of Medicine and School of Public Health, Kyoto University, Floor 2, Science Frontier Laboratory, Yoshida-konoe-cho, Sakyo-ku, Kyoto-shi, Kyoto, 606-8315 Japan; 4 Department of Oral Health Promotion, Graduate School of Medical and Dental Sciences, Tokyo Medical and Dental University, Tokyo, Japan; 5 Division for Regional Community Development, Liaison Center for Innovative Dentistry, Graduate School of Dentistry, Tohoku University, Sendai, Japan; 6 Institute for Future Initiatives, The University of Tokyo, Tokyo, Japan; 7 Japan Agency for Gerontological Evaluation Study (JAGES Agency), Tokyo, Japan

**Keywords:** Poverty, Oral health inequities, Public assistance, Free dental care access, Japan

## Abstract

**Background:**

Maintaining oral health is one of the global public health challenges. Income and out-of-pocket payments for dental care services are predictors of dental care utilisation. Although public assistance programmes guarantee income security for impoverished people, access barriers other than financial costs may cause unmet dental care needs. We aimed to explore the potential sociodemographic factors determining dental care utilisation among recipients of public assistance in Japan using linkage data of public assistance database and medical assistance claim data administered by municipalities.

**Methods:**

This was a retrospective cohort study involving a sample of public assistance recipients. We extracted the recipients’ sociodemographic data (age, sex, household number, employment status, nationality, disability certificates, and long-term care status) in January 2016 and observed them until December 2016 to identify incidences of dental care utilisation as outcomes. We performed a multivariable modified Poisson regression analysis with a robust standard error estimator to calculate the incidence ratio (IR) of dental care utilisation in each variable.

**Results:**

We identified a total of 4497 recipients at risk. Among them, 839 recipients used dental care services. Younger age was associated with a higher incidence of dental care utilisation. The female recipients had a higher incidence of dental care utilisation when compared to the male ones (adjusted IR, 1.22; 95% confidence interval [CI], 1.08–1.38). Immigrant recipients had a higher incidence of dental care utilisation than the Japanese ones (IR, 1.53; 95% CI, 1.16–2.01). Recipients with mental disabilities had higher incidences than those without disability certificates (IR, 1.30; 95% CI, 1.08–1.56).

**Conclusions:**

Non-financial sociodemographic inequities in dental care utilisation stemming from age, sex, nationality, and presence of mental disability were found despite minimum income protection and equitable financial dental service access amongst public assistance recipients in Japan. Providing targeted preventive care and treatments for dental care among underserved populations is required to tackle oral health inequities.

**Supplementary Information:**

The online version contains supplementary material available at 10.1186/s12939-021-01473-8.

## Background

Oral diseases (such as dental caries and periodontal diseases) include a range of chronic clinical conditions that affect the teeth and mouth [1, 2]. Although preventable, oral diseases are highly prevalent throughout life. Therefore, oral diseases are major global public health issues and have substantial adverse effects on an individual’s physical and mental health, further burdening the society [1–5].

As poverty is a strong determinant of oral health, governments in many countries have welfare programmes, which provide financial support to the poor, and the services include full or partial exemptions of dental care costs [6]. However, even though access to dental care services is ensured, previous studies have reported that other non-financial sociodemographic factors such as sex, marital status, race/ethnicity, nationality, education, job status, and social relationships are also associated with oral health [7–15]. In Japan, there is a governmental welfare programme called public assistance *‘seikatsu-hogo’* that is eligible for households living below the poverty line without any assets. Households on public assistance receive monthly minimum income benefits and are fully exempted from paying for dental care [16]. However, given the potential effects of non-financial socioeconomic statuses on poor oral health, the financial welfare programme may not fully compensate for the socioeconomic risks associated with poor oral health.

Nonetheless, to the best of our knowledge, no study has investigated the potential factors determining dental care utilisation among the impoverished population supported by the Japanese governmental welfare programme. Therefore, the purpose of this study was to explore the sociodemographic determinants of dental care utilisation among public assistance recipients using the linkage data of the municipal public assistance database and the medical assistance claim data in Japan.

## Methods

### Study design and participants

**Fig. 1:**
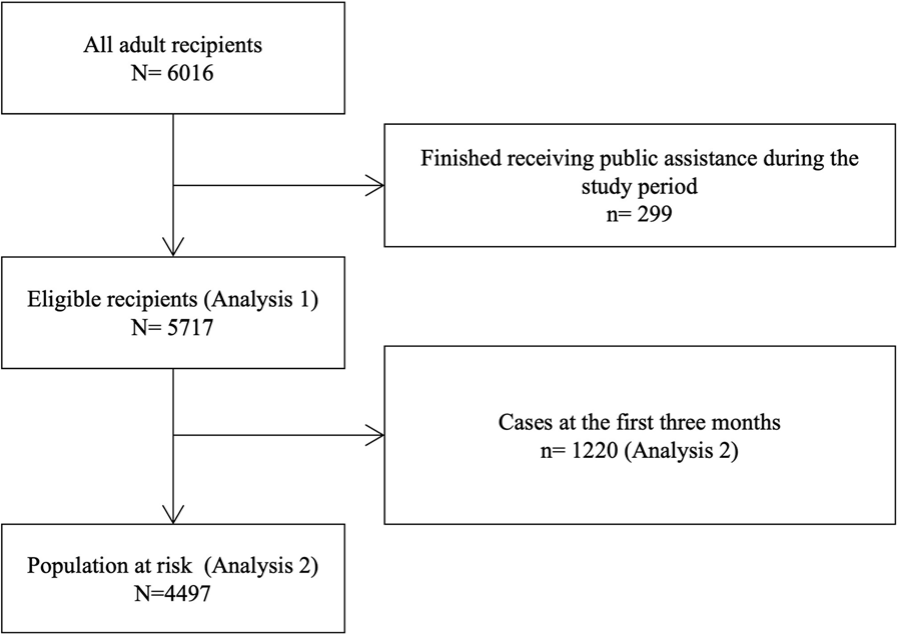
Flow-chart of study participants

This retrospective cohort study included all adults who received public assistance in two suburban municipalities, Osaka and Tokyo, in January 2016. In Japan, approximately 2% of the population receives public assistance [16]. Households eligible for public assistance receive monthly minimum income benefits and are fully exempted from paying for medical and dental care [16]. All the public assistance recipients could use the medical assistance programme (including payment for dental care) if they had medical-care vouchers (*iryo-ken*) from the welfare office. We excluded the data of participants who stopped receiving public assistance during the observational period, which occurred mainly due to participants’ increased incomes or deaths (Fig. 1).

### Data sources

For the baseline data, we used the public assistance recipients’ database from the welfare offices of two municipalities (A/B). In 2016, the population of municipality A was approximately 170,000 people, 23.7% of the population was over 65 years old, and 2.8% of the population was receiving public assistance. The financial index of municipality A was 1.1, and the unemployment rate was 4.3%. In contrast, the population of municipality B was approximately 100,000 people, 24.7% of the population was over 65 years old, and 1.8% of the population was receiving public assistance. The financial index of municipality B was 0.9, and the unemployment rate was 5.1%. The financial index is expressed as a ratio of total revenues to total expenditures in the municipality within three years; an index of less than one indicates that the municipality’s finances are in deficit. The average value of the financial index among all the Japanese municipalities was 0.50, which means the municipalities included in this study were relatively financially independent [17].

The public assistance database included information on age, sex, number of family members, household composition, nationality, working status, and income, including work income, pension, and disability pension. These data were collected by the staff of the municipality welfare offices to determine the utilisation of public assistance and the amount of monthly minimum income protection; thus, we did not have any missing data. To obtain the outcome data, we used medical assistance claim data between January, 2016 and December, 2016, which included dental claim data. The data comprised the recipients’ monthly medical and dental consultations, total consultation cost, total number of visits each month, and diagnoses.

Each municipality linked the two databases using individual identification codes. The welfare offices of the two municipalities agreed to provide anonymised data to the authors via a system company that provided the management software of the public assistance database to the municipalities. This study protocol was approved by the Ethics Committee of the Graduate School of Medicine of the University of Tokyo (Approval No: 11503).

### Measurements and variables

#### Outcome variables

From the medical assistance claim data, we identified the cumulative incidence of dental care utilisation, defined as receiving dental care services for the following oral diseases once or multiple times during the observational period: K00, disorders of tooth development and eruption; K01, embedded and impacted teeth; K02, dental caries; K03, other diseases of hard tissues of teeth; K04, diseases of pulp and periapical tissues; K05, gingivitis and periodontal diseases; and K06, other disorders of gingiva and edentulous alveolar ridge. These oral diseases were determined according to the International Classification of Disease, Tenth Edition (ICD-10) code [18]. These variables were considered as proxy measures of poor dental health conditions and accessibility to dental care services. We dichotomized the cumulative incidence of dental care utilisation into a binary variable exhibiting dental care utilisation or not.

#### Explanatory variables

Based on the data availability as of January 2016, we extracted the following information as demographic factors: age (continuous), sex (female or male), household composition (living alone or not), employment status (working or not), and nationality (Japanese or others). Since recipients of disability assistance can receive additional income and social care benefits, we considered mental, intellectual, and physical disabilities as potential confounding factors by noting information on the qualifications for welfare benefits for disabled people, as municipality officials certify them with the diagnosis given by designated physicians. We also adjusted for the levels of long-term care needs based on the information in the public long-term care insurance system. In the system, there are seven nationally standardised levels of long-term care need (support requirement levels 1 and 2, and care need levels 1 to 5) [16]. People aged 65 years and above who are potentially in need of long-term care and people aged 40 years and above with the designated diseases can apply for insurance benefits. Based on the definition of the level, insurers (in most cases, municipality governments) assess and certify the insurance benefits. The benefits provided to the recipient are determined based on the certified level. In this study, we classified the levels into ‘support requirement’ level and ‘care need’ level. We coded municipality as a dummy variable to adjust for the cluster structures and unmeasured cultural and environmental characteristics of the two municipalities (A/B).

### Statistical analysis

First, we described the characteristics of all the study participants and recipients who received dental care during the observation period. Second, we performed a univariable modified Poisson regression analysis which can estimate relative risks of each explanatory variable on binary outcome variable [19], and calculated the crude cumulative incidence ratio (IR) of dental care utilisation and 95% confidence interval (CI) of each explanatory variable. Third, we performed a multiple modified Poisson regression analysis to calculate the multivariable-adjusted IR of each explanatory variable (Analysis 1). To identify the incidence of dental care service utilisation amongst the population at risk, we performed an additional analysis, which excluded recipients who had received dental care in the first three months (Analysis 2). This allowed us to exclude recipients who used dental care services regularly for preventive and treatment measures because their dental care utilisation would have occurred frequently during these three months [20, 21]. Consequently, we could verify the actual incidence of dental care utilisation among the study participants. Furthermore, we performed two sensitivity analyses. To confine dental care utilisation only for treatment purposes, we limited our outcome variables to the incidence of dental caries using ICD-10 code of K02 (Analysis S1). Since dental care utilisation for preventive measures was also reported to occur over six months [20, 21], we performed an additional analysis that excluded recipients who had received dental care in the first six months (Analysis S2). The robust standard error estimator was adopted for all statistical analyses to calculate 95% CIs. All the analyses were performed using STATA SE Ver.16.2 (Stata Corp., College Station, TX, USA).

## Results

We obtained the data of 6016 people on public assistance, 4497 recipients did not use dental care during the first three months of the observational period (Fig. 1). Among them, 2281 (50.7%) were women, 859 (19.0%) had jobs, 392 (8.7%) had mental disabilities, and 124 (2.8%) were immigrants. Regarding the recipients’ oral health, 839 (18.7%) utilised dental care during the observational period (Table 1, Figure S1). The univariable modified Poisson regression analysis showed that recipients who were young, women, immigrants, employed, and certified with mental disabilities had higher incidences of dental care utilisation (Table S1).

**Table 1:** Characteristics of the study participants and the recipients on dental care services

Character	Category	Analysis 1	Participants on dental care	Analysis 2	Participants on dental care
		Total participants		Population at risk	
		(*N* = 5717)	(*n* = 2059)	(*N* = 4497)	(*n* = 839)
		N (%)	n, % for N	N (%)	n, % for N
Age	Mean (SD)	62.6 (15.9)	60.2 (15.8)	63.0 (15.9)	59.2 (15.6)
Sex	Male	2790 (48.8)	950, 34.1%	2216 (49.3)	376, 17.0%
	Female	2927 (51.2)	1109, 37.9%	2281 (50.7)	463, 20.3%
Living alone	Yes	3762 (65.8)	1353, 36.0%	2943 (65.4)	534, 18.1%
	No	1955 (34.2)	706, 36.1%	1554 (34.6)	305, 19.6%
Working status	Yes	1088 (19.0)	433, 39.8%	856 (19.0)	201, 23.5%
	No	4629 (81.0)	1626, 35.1%	3641 (81.0)	638, 17.5%
Nationality	Japanese	5558 (97.2)	1986, 35.7%	4373 (97.2)	801, 18.3%
	Other	159 (2.8)	73, 45.9%	124 (2.8)	38, 30.6%
Long-term care status	None	4896 (85.6)	1762, 36.0%	3870 (86.1)	736, 19.0%
	Support required	202 (3.5)	62, 30.7%	163 (3.6)	23, 14.1%
	Care needs	619 (10.8)	235, 38.0%	464 (10.3)	80, 17.2%
Disabilities certificate	None	4645 (81.2)	1605, 34.6%	3709 (82.5)	669, 18.0%
	Mental disability	536 (9.4)	246, 45.9%	392 (8.7)	102, 26.0%
	Intellectual disability	83 (1.5)	33, 39.8%	59 (1.3)	9, 15.3%
	Physical disability	453 (7.9)	175, 38.6%	337 (7.5)	59, 17.5%
Municipality	A	4213 (73.7)	1537, 36.5%	3310 (73.6)	634, 19.2%
	B	1504 (26.3)	522, 34.7%	1187 (26.4)	205, 17.3%

Analysis 1 includes all eligible participants, and Analysis 2 includes population at risk after excluding cases at the first three months. *SD* Standard Deviation

In the multivariable modified Poisson regression analyses, the results of Analysis 1 showed that recipients who were young, women, immigrants, certified with mental disabilities, certified with ‘care-needs’ level of long-term care, and those who lived alone had high incidences of dental care utilisation (Table 2). The results of Analysis 2 showed that recipients who were young (IR, 0.87 (by 10-year-age groups); 95% CI, 0.84–0.91), women (IR, 1.22; 95% CI, 1.08–1.38), immigrants (IR, 1.53; 95% CI, 1.16–2.01), and certified with mental disabilities (IR, 1.30; 95% CI, 1.08–1.56) were associated with high incidences of dental care utilisation. Recipients, who lived alone, were employed, and who needed long-term care had a slightly high incidence of dental care utilisation (Table 2). Our sensitivity analysis (Analysis S1) showed that recipients who were women and certified with mental disabilities were associated with high incidences of dental caries (IR, 1.30; 95% CI, 1.14–1.49 and IR, 1.32; 95% CI, 1.09–1.59; respectively); however, there was no strong association between nationality and dental caries diagnosis (Additional File 2). Moreover, the results of Analysis S2 that excluded users from dental services in the first six months showed similar results as that of Analysis 2 (Additional File 3).

**Table 2:** Adjusted incidence ratios for the incidence of dental care utilization among public assistance recipients

		Analysis 1	Analysis 2
		IR, (95% CI)	IR, (95% CI)
Age	by 10 year	0.90 (0.88–0.93)	0.87 (0.84–0.91)
Sex	Male	Ref	Ref
	Female	1.14 (1.06–1.22)	1.22 (1.08–1.38)
Working status	No	Ref	Ref
	Yes	1.03 (0.94–1.13)	1.15 (0.99–1.34)
Living alone	No	Ref	Ref
	Yes	1.08 (1.00–1.16)	1.06 (0.93–1.21)
Nationality	Japanese	Ref	Ref
	Other	1.25 (1.05–1.49)	1.53 (1.16–2.01)
Long-term care status	None	Ref	Ref
	Support required	0.99 (0.80–1.23)	0.94 (0.64–1.40)
	Care needs	1.26 (1.12–1.41)	1.20 (0.95–1.50)
Disabilities certificate	None	Ref	Ref
	Mental disability	1.22 (1.10–1.36)	1.30 (1.08–1.56)
	Intellectual disability	0.98 (0.75–1.29)	0.65 (0.36–1.19)
	Physical disability	1.11 (0.98–1.26)	1.02 (0.80–1.31)
Municipality	A	Ref	Ref
	B	0.94 (0.87–1.02)	0.91 (0.79–1.06)

Analysis 1 includes all eligible participants, and Analysis 2 includes population at risk after excluding cases at the first three months. *IR* Incidence Ratio, *CI* Confidence Interval

## Discussion

Our study found that among public assistance recipients in Japan, the incidence of dental care utilisation was high among young people, women, immigrants, and those with mental disability certificates. This was the first study that demonstrated sociodemographic inequities in dental care utilisation among adult public assistance recipients whose dental care utilisations and minimum incomes were financially ensured. The strength of this study was that by using existing standardised databases without missing data, we identified the inequities in dental care utilisation among public assistance recipients who are usually difficult to reach through standard social surveys.

### Findings in context

The high incidence of dental care utilisation among the younger recipients observed in this study can be attributed to the following factors. First, older people who have lost their teeth may not perceive the need for dental care services, except for dentures [22]. Second, younger recipients might have higher oral health literacy and accessibility to dental care than older recipients. The findings that the female recipients and those with mental disabilities had a higher incidence of dental care utilisation were consistent with the results of other recent studies [23–25]. Women may have a greater preference for maintaining oral health and aesthetic appearance than men due to the societal norms [23–25], resulting in their higher use of dental care services than men. It is conceivable that we might have overestimated the usage of dental care services among female recipients because our outcome depended on recipients’ dental care utilisations. Moreover, the reduced motivation of patients with mental disabilities or severe mental health disorders to care for their oral health may explain the association between mental disability and poor oral health [26, 27]. Reduced protective factors for oral diseases as side effects of medications (such as decreased saliva secretion) can also explain the association [28].

There may be several possible explanations for the higher incidence of dental care utilisation among immigrants than the Japanese recipients. First, free dental care associated with the public assistance programme might have inflated immigrants’ dental care attendance, as payment for dental services may be needed in their own countries. Second, our findings were consistent with those of a recent study on non-recipient immigrants. Oral diseases and dental care utilisation were more prevalent among immigrants than the Japanese [29, 30]. Baseline disease levels may be worse among immigrant recipients of public assistance than among Japanese recipients. Third, immigrant recipients of public assistance may also use more dental care services for preventive measures than for treatment purposes. Our sensitivity analysis that showed the absence of a strong association between nationality and dental care utilisation for the treatment of dental caries underscored this hypothesis. The frequency of dental visits for symptoms of dental caries may be similar between Japanese and immigrant recipients. In this context, Japanese recipients might be underserved by dental care services for preventive measures. Finally, immigrant recipients have also been reported to use medical care services more frequently than Japanese recipients [31]. Immigrant recipients may experience social isolation, which may lead to their increased demands for support by healthcare professionals, including dental care providers. Further studies are required to examine the reasons for high incidences of dental visits among immigrants.

Although this study could not identify the reason for not using dental care services, according to the national survey of dental diseases, approximately 30% of Japanese adults have dental diseases that require treatment [22]. Considering that the study population faces poverty that is related to poor oral health [13], there may be a larger proportion of people who did not receive dental care despite their unpreferable oral health conditions among the public assistance recipients. Recipients who did not use dental care services in this study may have underutilised dental care.

### Practice and policy implications

Our study provides novel evidence that sociodemographic inequities in dental care utilisation are still prevalent among public assistance recipients in Japan, even though they are assured free dental care access and minimum income by the government welfare programme. Individual socioeconomic backgrounds may be involved in determining dental care utilisation. Since oral health strongly influences individual and societal burdens, identifying population segments at risk of underserved dental care and prioritising preventive and treatment activities may be effective [32, 33]. Dental care providers and policymakers should also consider designing dental care interventions using population-based approaches. Although the Japanese government will implement a mandated health management programme for public assistance recipients in 2021 [34], this programme does not include oral healthcare. Thus, the government also needs to provide an additional oral health management programme to public assistance recipients.

### Limitations

Our study had several limitations. First, although we used longitudinal data, there was still the possibility of reverse causation. Some people may have developed severe oral diseases and other illnesses, which would have resulted in financial difficulties and the subsequent need for public assistance. Nonetheless, the results of the analyses that removed the incidence of dental care utilisation during the first three months minimised the risk of reverse causation. Second, our use of medical assistance claim data might have over- or under-estimated the incidence of dental care utilisation. The incidence was only evaluated for utilisation of dental care services; thus, recipients who experienced severe health conditions or social isolation might not have been able to access dental care services, which may have resulted in differential misclassification. Third, there were important factors that were not evaluated in this study, such as the severity of diseases and what the dental treatments entailed, and this may have potentially biased our findings. For example, we could not differentiate between dental services used for preventive or treatment purposes from the data. Finally, since this study used data from only two municipalities in Japan, the generalisability of our findings to other populations may be limited.

## Conclusion

Our study suggests that public assistance recipients who are young, female, immigrants, and have mental disabilities have a high incidence of dental care utilisation. Sociodemographic inequities in the use of dental care services exist despite free access to dental services and minimum income protection amongst public assistance recipients in Japan. Providing targeted preventive and curative dental care for underserved populations is required. Further investigations using detailed information about broader sociodemographic factors, the severity of the oral health conditions, and dental treatments received are warranted.

## Supplementary Information

**Additional file 1: Table S1**. Crude incidence ratios (IR) and 95% confidence intervals (CI) for the incidence of dental care utilisation among public assistance recipients.

**Additional file 2: Table S2**. Adjusted incidence ratios (IR) and 95% confidence intervals (CI) for the incidence of dental caries diagnosis among public assistance recipients (Analysis S1).

**Additional file 3: Table S3**. Adjusted incidence ratios (IR) and 95% confidence intervals (CI) for incidence of the dental care utilisation excluding cases at the first six months.

**Additional file 4: Figure S1**. Distribution of the cumulative incidence of dental care utilisation among public assistance recipients.

## Data Availability

The data used in this study were obtained from the participating municipalities in Japan; however, there are restrictions regarding the availability of these data, which were used under licence for the current study, and are not publicly available. The data are available from the authors upon reasonable request, with the permission of the municipalities.

## Abbreviations

IR: Incidence ratio
CI: Confidence interval
ICD-10: International Classification of Disease, Tenth Edition
