# Supplementary material for: Sociodemographic inequities in dental care utilisation among governmental welfare recipients in Japan: a retrospective cohort study
Source: Int J Equity Health. 2021 Jun 16;20:141. doi: 10.1186/s12939-021-01473-8 (PMC8207738; doi:10.1186/s12939-021-01473-8)
Supplement: Supplementary file 1 — Additional file 1: Table S1. Crude incidence ratios (IR) and 95% confidence intervals (CI) for the incidence of dental care utilisation among public assistance recipients. [file 12939_2021_1473_MOESM1_ESM.docx]

**Additional File 1**

| Table.S1 Crude incidence ratios (IR) and 95% confidence intervals (CI) for the incidence of dental care utilization among public assistance recipients | | | | |
| --- | --- | --- | --- | --- |
|  |  |  |  |  |
|  |  |  | Analysis 1 | Analysis 2 |
|  |  |  | IR, (95% CI) | IR, (95% CI) |
| Age | by 10 year |  | 0.91 (0.90- 0.93) | 0.87 (0.84- 0.90) |
| Sex |  |  |  |  |
|  | Male |  | Ref | Ref |
|  | Female |  | 1.11 (1.04- 1.19) | 1.20 (1.06- 1.35) |
| Working status | |  |  |  |
|  | No |  | Ref | Ref |
|  | Yes |  | 1.13 (1.04- 1.23) | 1.34 (1.17- 1.54) |
| Living alone |  |  |  |  |
|  | No |  | Ref | Ref |
|  | Yes |  | 1.00 (0.93- 1.07) | 0.92 (0.81- 1.05) |
| Nationality |  |  |  |  |
|  | Japanese |  | Ref | Ref |
|  | Other |  | 1.28 (1.08- 1.53) | 1.67 (1.27- 2.20) |
| Long-term care status | |  |  |  |
|  | None |  | Ref | Ref |
|  | Support required |  | 0.85 (0.69- 1.05) | 0.74 (0.51- 1.09) |
|  | Care needs |  | 1.05 (0.95- 1.17) | 0.91 (0.74- 1.12) |
| Disabilities certificate | |  |  |  |
|  | None |  | Ref | Ref |
|  | Psychological disability |  | 1.33 (1.20- 1.47) | 1.44 (1.20- 1.73) |
|  | Intellectual disability |  | 1.15 (0.88- 1.50) | 0.85 (0.46- 1.55) |
|  | Physical disability |  | 1.12 (0.99- 1.26) | 0.97 (0.76- 1.24) |
| Municipality |  |  |  |  |
|  | A |  | Ref | Ref |
|  | B |  | 0.95 (0.88- 1.03) | 0.90 (0.78- 1.04) |
| Analysis 1 includes all eligible participants, Analysis 2 includes population at risk after excluding cases at the first three months | | | | |
