# Supplementary material for: Sociodemographic inequities in dental care utilisation among governmental welfare recipients in Japan: a retrospective cohort study
Source: Int J Equity Health. 2021 Jun 16;20:141. doi: 10.1186/s12939-021-01473-8 (PMC8207738; doi:10.1186/s12939-021-01473-8)
Supplement: Supplementary file 2 — Additional file 2: Table S2. Adjusted incidence ratios (IR) and 95% confidence intervals (CI) for the incidence of dental caries diagnosis among public assistance recipients (Analysis S1). [file 12939_2021_1473_MOESM2_ESM.docx]

**Additional File 2**

| Table.S2 Adjusted incidence ratios (IR) and 95% confidence intervals (CI) for the incidence of dental caries diagnosis among public assistance recipients (Analysis S1) | | | | |
| --- | --- | --- | --- | --- |
|  |  |  |  |  |
|  |  |  | All eligible participants | After excluding cases at the first three months |
|  |  |  | IR, (95% CI) | IR, (95% CI) |
| Age | by 10 year |  | 0.86 (0.83- 0.89) | 0.87 (0.83- 0.90) |
| Sex |  |  |  |  |
|  | Male |  | Ref | Ref |
|  | Female |  | 1.21 (1.10- 1.34) | 1.30 (1.14- 1.49) |
| Working status | |  |  |  |
|  | No |  | Ref | Ref |
|  | Yes |  | 1.04 (0.92- 1.16) | 1.10 (0.94- 1.29) |
| Living alone |  |  |  |  |
|  | No |  | Ref | Ref |
|  | Yes |  | 1.02 (0.92- 1.13) | 1.06 (0.92- 1.22) |
| Nationality |  |  |  |  |
|  | Japanese |  | Ref | Ref |
|  | Other |  | 0.93 (0.70- 1.24) | 0.99 (0.69- 1.43) |
| Long-term care status | |  |  |  |
|  | None |  | Ref | Ref |
|  | Support required | | 0.82 (0.59- 1.14) | 0.85 (0.56- 1.29) |
|  | Care needs |  | 0.78 (0.63- 0.97) | 0.81 (0.62- 1.07) |
| Disabilities certificate | |  |  |  |
|  | None |  | Ref | Ref |
|  | Psychological disability | | 1.21 (1.05- 1.40) | 1.32 (1.09- 1.59) |
|  | Intellectual disability | | 0.85 (0.58- 1.25) | 0.73 (0.41- 1.30) |
|  | Physical disability | | 1.05 (0.86- 1.27) | 1.05 (0.82- 1.36) |
| Municipality |  |  |  |  |
|  | A |  | Ref | Ref |
|  | B |  | 0.84 (0.75- 0.95) | 0.82 (0.71- 0.96) |
|  | | | | |
