# Supplementary material for: Sociodemographic inequities in dental care utilisation among governmental welfare recipients in Japan: a retrospective cohort study
Source: Int J Equity Health. 2021 Jun 16;20:141. doi: 10.1186/s12939-021-01473-8 (PMC8207738; doi:10.1186/s12939-021-01473-8)
Supplement: Supplementary file 3 — Additional file 3: Table S3. Adjusted incidence ratios (IR) and 95% confidence intervals (CI) for incidence of the dental care utilisation excluding cases at the first six months. [file 12939_2021_1473_MOESM3_ESM.docx]

**Additional File 3**

| Table.S3 Adjusted incidence ratios (IR) and 95% confidence intervals (CI) for incidence of the dental care utilization excluding cases at the first six months. | | | |
| --- | --- | --- | --- |
|  |  |  |  |
|  |  |  | Analysis S2 |
|  |  |  | IR, (95% CI) |
| Age | by 10 year |  | 0.90 (0.85- 0.95) |
| Sex |  |  |  |
|  | Male |  | Ref |
|  | Female |  | 1.26 (1.06- 1.50) |
| Working status | |  |  |
|  | No |  | Ref |
|  | Yes |  | 1.02 (0.82- 1.28) |
| Living alone |  |  |  |
|  | No |  | Ref |
|  | Yes |  | 1.11 (0.92- 1.33) |
| Nationality |  |  |  |
|  | Japanese |  | Ref |
|  | Other |  | 1.66 (1.11- 2.48) |
| Long-term care status | |  |  |
|  | None |  | Ref |
|  | Support required | | 0.80 (0.46- 1.40) |
|  | Care needs |  | 1.14 (0.83- 1.55) |
| Disabilities certificate | |  |  |
|  | None |  | Ref |
|  | Psychological disability | | 1.26 (0.95- 1.65) |
|  | Intellectual disability | | 0.71 (0.30- 1.64) |
|  | Physical disability | | 0.92 (0.64- 1.32) |
| Municipality |  |  |  |
|  | A |  | Ref |
|  | B |  | 0.84 (0.68- 1.04) |
