# Supplementary material for: Sociodemographic inequities in dental care utilisation among governmental welfare recipients in Japan: a retrospective cohort study
Source: Int J Equity Health. 2021 Jun 16;20:141. doi: 10.1186/s12939-021-01473-8 (PMC8207738; doi:10.1186/s12939-021-01473-8)
Supplement: Supplementary file 4 — Additional file 4: Figure S1. Distribution of the cumulative incidence of dental care utilisation among public assistance recipients. [file 12939_2021_1473_MOESM4_ESM.docx]

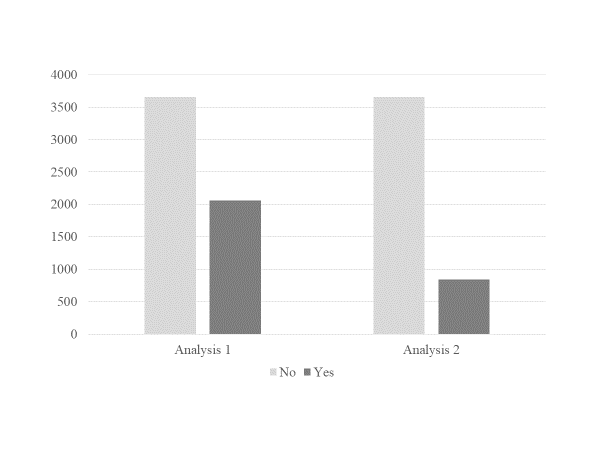
Figure S1. Distribution of the cumulative incidence of dental care utilisation among public assistance recipients.

Analysis 1 included all eligible participants, and Analysis 2 included population at risk after excluding cases at the first three months.
